# Divergent Chemosymbiosis-Related Characters in *Thyasira* cf. *gouldi* (Bivalvia: Thyasiridae)

**DOI:** 10.1371/journal.pone.0092856

**Published:** 2014-03-21

**Authors:** Rebecca T. Batstone, Jason R. Laurich, Flora Salvo, Suzanne C. Dufour

**Affiliations:** Department of Biology, Memorial University of Newfoundland, St. John's, Newfoundland, Canada; University of Vienna, Austria

## Abstract

Within the marine bivalve family Thyasiridae, some species have bacterial chemosymbionts associated with gill epithelial cells while other species are asymbiotic. Although the abundance of symbionts in a particular thyasirid species may vary, the structure of their gills (i.e., their frontal-abfrontal thickening) does not. We examined gill structure in a species tentatively identified as *Thyasira gouldi* from a Northwest Atlantic fjord (Bonne Bay, Newfoundland) and found remarkable differences among specimens. Some individuals had thickened gill filaments with abundant symbionts, while others had thin filaments and lacked symbionts. We could differentiate symbiotic and asymbiotic specimens based on the size and outline of their shell as well as 18S rRNA, 28S rRNA and CO1 sequences. The wide morphological, genetic and symbiosis-related disparity described herein suggests that chemosymbiosis may influence host divergence, and that *Thyasira gouldi* forms a cryptic species complex.

## Introduction

The Thyasiridae (superfamily Thyasiroidea [1]) is one of five bivalve families that have established symbiotic relationships with chemoautotrophic bacteria [2]. Not all species within the family are chemosymbiotic [3], [4], and among symbiotic species, nutritional reliance on symbionts varies [5]. In all but one chemosymbiotic thyasirid species, the bacterial symbionts are extracellular [3], [6], residing either in enlarged spaces limited by the microvilli and the cell membrane in the bacteriocyte zone of modified, abfrontally expanded gill filaments, or among the microvilli of abfrontal epithelial cells in gills with shorter filaments [4]. Ultrastructural evidence indicates that thyasirid gills periodically engulf and digest their symbionts [3], [5], [7], [8], and there is no evidence for the direct transfer (“milking”) of nutrients from extracellular symbionts to thyasirids. Relationships between thyasirids and their extracellular symbionts may be relatively less specific or complex than in other groups where symbionts are intracellular.

Symbiont presence in the Thyasiridae has been linked to gill structure [4], and a recent molecular phylogeny suggests that symbiotic species belong to more than one distinct clades within the family [1]. One well-supported clade consists of *Thyasira flexuosa* Montagu, 1803, *T. gouldi* Philippi, 1845 and *T. polygona* Jeffreys, 1864, species with abfrontally thickened gills typically containing large numbers of symbionts [3], [4], [9]. Both *T. flexuosa* and *T. gouldi* reportedly have large geographic ranges [10], [11], and specimens examined from various sites show similar features and abundant symbionts [3], [4]. Although symbiont abundance can change according to particulate food availability [9], the structure of chemosymbiotic bivalve gills, i.e., the degree of frontal-abfrontal elongation of filaments, or ‘gill type’ [4], has not been shown to vary along with symbiont abundance within a host species.

*Thyasira gouldi* is considered to be a panarctic species, usually found in organically enriched clay-grade sediments at < 50 m depth [11]–[13]. *T. gouldi* was initially collected in deep water off Massachusetts [14], [15], and has been sampled from various cold water estuarine and fjord sites, including Scottish Sea Lochs, Southern Norwegian fjords, and the Southwest coast of the United Kingdom [11], [16]. We recently sampled thyasirids, tentatively identified as *T. gouldi* (hereafter referred to as *T*. cf. *gouldi*) from a fjord in Bonne Bay, Newfoundland, Canada. The Bonne Bay specimens share shell characteristics with *T. gouldi* described from the eastern and western Atlantic [11], [15], and resemble described *T. gouldi* specimens from the eastern Atlantic in main features of their internal anatomy [11] – we are unaware of descriptions of the internal anatomy for western Atlantic specimens.

Here, we report striking differences in symbiont presence and gill filament morphology among *Thyasira cf. gouldi* specimens from Bonne Bay, where many individuals contain thickened gills with abundant symbionts while others have thin gill filaments that lack symbionts. We hypothesize that symbiotic and asymbiotic specimens also differ in other characters (size, internal anatomy, shell shape, prodissoconch size, and partial 18S rRNA, 28S rRNA and CO1 gene sequences), and form more than one morphologically and genetically distinct groups.

## Materials and Methods

### Thyasirid sampling

Permits to collect invertebrates from Bonne Bay for experimental purposes were obtained from Fisheries and Oceans Canada. Thyasirids were sampled from Bonne Bay (49°30′N 57°55′W), a fjord partially separated from the Gulf of St. Lawrence by a 50 m deep sill retaining a deep layer of cold water year round [17]. Using a Peterson grab, we collected specimens from three sites within East Arm: Southeast Arm (S, 49°27′51.46″N, 57°43′09.04″W, 30 m depth), Deer Arm (D, 49°32′43.48″N, 57°50′28.45″W, 30 m depth), and Neddy's Harbour (N, 49°31′21.44″N, 57°52′11.07″W, 15 m depth) in May and August 2010, and April, June, October, and December 2011. Sediments were sieved (1 mm mesh) to retrieve thyasirids, and 152 specimens are analysed herein. We carefully removed the gills from each specimen, keeping one gill for morphological analyses (N = 124), molecular analysis (N = 104), or another purpose. We obtained overlapping morphological and molecular data for 76 of the 152 specimens.

The shell length (anterior-posterior) was recorded for all specimens. We compared the shell length of symbiotic and asymbiotic thyasirids (following morphological and molecular confirmation; N = 76) using LS analysis in JMP; no major violations of assumptions were found based on the diagnostic residual plots observed. The valves of a subset of individuals (N = 37) were retained for shell shape analysis and prodissoconch measurements.

### Light and Transmission Electron Microscopy of Gills

Gills retained for morphological analysis (N = 124) were fixed in 2.5% gluteraldehyde in 0.1 M sodium cacodylate buffer for 24 hours, post-fixed in 1% osmium tetroxide in the same buffer for 1 h, dehydrated in an ascending ethanol series, and embedded in EPON resin. Semi-thin (1 μm) sections were made using a LKG Bromma 8800 ultramicrotome, and stained with 1% toluidine blue in 1% sodium borate for light microscopy. Ultra-thin sections (60 nm) of each gill were post-stained with uranyl acetate and lead citrate and observed using a Philips 300 transmission electron microscope.

### Shell Shape Analysis and Prodissoconch Measurement

To test for differences in shell outline among specimens, we performed statistical analyses of Elliptic Fourier coefficients, landmark and size independent descriptors of shapes that are particularly suited for comparisons of bivalve shells [18]. The left valve of 37 individuals was imaged using a dissecting microscope. Outlines of each shell were digitized using the tool E-Snake [19] in ImageJ [20] and analyzed using SHAPE 1.3a [21]. Each shell outline was normalized for size and rotated using the umbo as reference, and Elliptic Fourier coefficients (EFCs) were then determined for each shell. A Principal Components Analysis (PCA) of the variance-covariance matrix was run to summarize the shape variation based on EFCs [18], and a graph produced to show the distribution of individual shell shapes along the first two principal components. Reconstructions of the shell contours at each extremity of the first two significant axes of the PCA were then made. PCA matrix coordinates (from significant axes) were used to compare the different shells in a cluster analysis in Primer 6.0 [22] based on Euclidian distance. A first cluster analysis was run based on the 10 first harmonics to discern individuals based on general differences in shape, and a t-test in Statistica was used to compare shell lengths between isolated groups. A second PCA and cluster analysis was performed based on one of the groups of shells identified by the first cluster analysis, using the 20 first harmonics (without the first) to decrease the importance of the general outline of the shell (described by the first harmonic) and therefore emphasize finer differences in outline [18]. An ANOSIM (999 permutations) was performed in Primer 6.0 on the second cluster to investigate OTU groupings.

We imaged the umbonal region of the valves retained for shape analysis and measured prodissoconch length (to 0.1 mm accuracy) using a measuring tool in ImageJ [20], and used a t-test in Statistica to compare the prodissoconch length of symbiotic and asymbiotic individuals. Because the prodissoconch of some specimens was damaged, we report measurements from 25 of those 37 valves.

### DNA extraction, PCR amplification and sequencing

Considering that there are available 18S and 28S rRNA gene sequences of *Thyasira gouldi* from Mill Bay, Salcombe, UK [23] and Firth of Forth, UK [24], we chose to sequence this gene in the Bonne Bay specimens. These nuclear genes are useful in phylogenetic studies at various systematic scales because they contain both variable and highly conserved regions [25]. In addition, we also sequenced the more rapidly evolving CO1 gene, often used for species barcoding, although there are no available *T. gouldi* CO1 sequences in GenBank.

Gills (N = 104) were individually kept in 95% ethanol upon dissection. We isolated and purified DNA using the QIAgen DNeasy Blood and Tissue kit, following the spin-column protocol for animal tissues.

Polymerase chain reactions (PCRs) were performed using gene-specific sets of primers: 1) 18S-5′ [26] and 18S1100R [27] for a ∼1000 base pair (bp) fragment of the 18S rRNA gene; 2) LSU-5′[28] and LSU1600R [27] for a ∼1500 bp fragment of the 28S rRNA gene; and 3) BivF4_t1 and BivR1_t1 [29] for a 667 bp fragment of the CO1 gene. For the nuclear genes, we performed 25 μL PCR using the Promega PCR Master Mix (Promega Corp.) containing 1 μL of template DNA, 50 μL/mL Taq DNA polymerase, 400 μM of each dNTP, 3.0 and 2.5 mM of MgCl_2_ for 18S and 28S, respectively, and reaction buffer at a pH of 8.5. Thermocycling was run as follows: 4 min of initial denaturation at 94°C, followed by 35 cycles at 94°C for 30 s, 30 s at annealing temperatures of 54°C and 52°C for 18S and 28S, respectively, 2 min at 72°C, with a final elongation at 72°C for 5 min. Products filtered using Acro-Pro 100K-Omega filters (Pall Life Sciences) were prepared for sequencing using BigDye Terminator v 3.1 Cycle Sequencing Ready Reaction Mix (Applied Biosystems) and electrophoresed on an Applied Biosystems 3730 DNA Analyzer automated capillary sequencer. Amplification and sequencing of the CO1 gene was performed as in [29].

### Sequence analyses

We aligned and compared forward and reverse sequences using Sequencher (v. 5.0, Gene Codes Corp.) and used Basic Local Alignment Search Tool (BLAST [30]) to find closely related sequences in GenBank. Operational taxonomic units (OTUs) were identified using MEGA5 [31] by aligning and then grouping individual 18S and 28S thyasirid sequences based on polymorphic nucleotide sites; sequences were therefore identical within OTUs (i.e., no base discrepancies excluding ambiguous sites). All alignments were executed with default values in MEGA5, using ClustalW [32]. We identified and removed poorly aligned sites using Gblocks v. 0.91b [33].

We determined the relatedness within and between our OTU pairs by calculating the average evolutionary divergence (*d*) for CO1 fragments by constructing a distance matrix using “*p*-distance” as the evolutionary model in MEGA5; thus, the proportion of base discrepancies could be compared between each pair of OTUs, accounting for fragment size. We used 2000 bootstrap replicates to generate standard error values for each distance comparison, and treated gaps or missing data with pairwise deletion. To determine whether the calculated *d* differed within and between OTUs, we conducted a one-way ANOVA in RStudio (R v. 3.0.0). Tukey's HSD post-hoc tests were used to determine which group comparisons were significantly different.

## Results

### Anatomical characters and size

**Figure 1:**
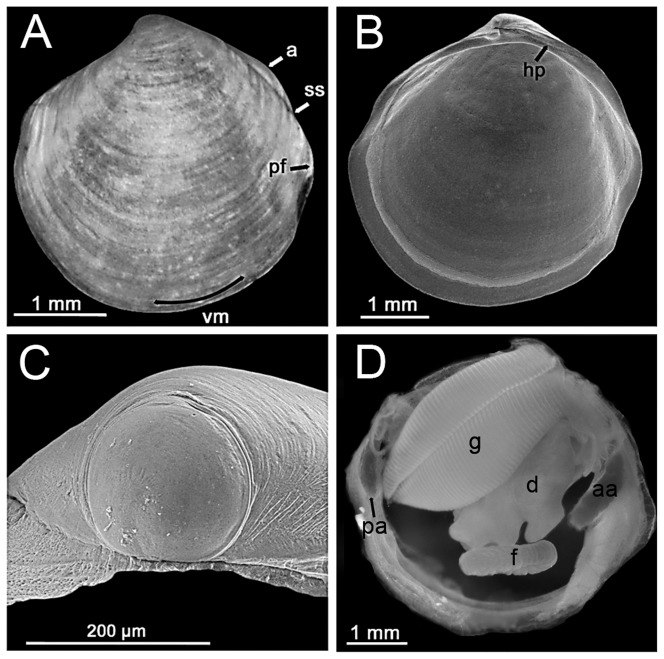
Morphology of *Thyasira* cf. *gouldi* from Bonne Bay, Newfoundland. **A.** Outer view of the left shell valve, with a weakly projecting auricle (a), a well-defined submarginal sulcus (ss) forming a marginal sinus, a distinct, yet rounded, posterior fold (pf), and a rounded ventral margin (vm). **B.** Inner view of the right valve, revealing the absence of dentition along the hinge plate (hp). **C.** Scanning electron micrograph of the larval shell (∼ 181 μm diameter). **D.** Internal anatomy, showing a gill (g) with two demibranchs, the digestive diverticula (d), foot (f), anterior adductor (aa), posterior adductor (pa) and mantle margin, thickened at the anterior end.

The shells of all specimens were generally equilateral-ovate, slightly higher than long, and bisinuate (Fig. 1a,b); shell dorso-ventral length and antero-ventral curvature slightly varied among specimens. A weakly projecting auricle defined the posterior region, with a submarginal sulcus forming a marginal sinus and a posterior fold forming a posterior sinus. The hinge plate lacked a clear cardinal tooth (Fig. 1b). Many individuals had patches of rust-coloured deposits on the anterior and posterior ends of their shells. Prodissoconch length ranged between 180 and 210 μm (N = 25; Fig. 1c).

In all specimens, gills had two demibranchs, the mantle margin was thickened at the anterior end, the foot was elongate and vermiform, and digestive diverticula formed a single mass (i.e., not as branched as in other thyasirid species) (Fig. 1d). The size range of all individuals we retrieved on the 1 mm mesh was between 1.8 – 5.0 mm.

### Gill morphology, symbiont presence, and corresponding characters

**Figure 2:**
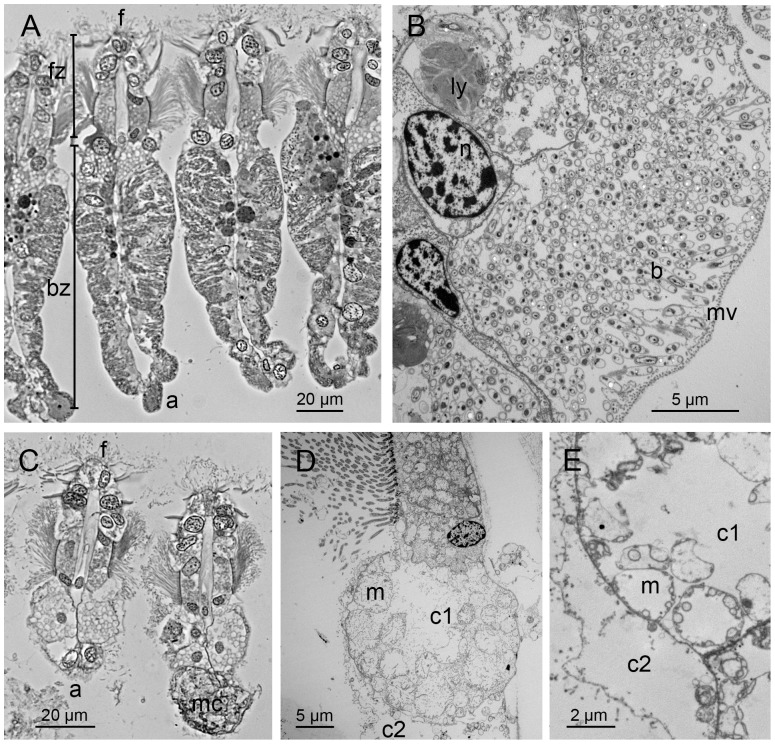
Light and electron micrographs of gill filaments of *Thyasira* cf. *gouldi*. **A.** Symbiotic specimen. Light micrograph of a semi-thin, transverse section through four gill filaments. The ciliated frontal zone (fz) and bacteriocyte zone (bz) are highlighted. a: abfrontal end of a filament; f: frontal end of a filament. **B.** Symbiotic specimen. TEM of cells in the bacteriocyte zone of a gill filament, showing abundant bacteria (b), maintained extracellularly in pockets limited by extensions of host cell cytoplasm bearing microvilli (mv). Nuclei (n) and lysed remains of digested symbionts (ly) are visible in the host cell cytoplasm. **C.** Asymbiotic specimen. Light micrograph of a semi-thin, transverse section through two gill filaments. Note the shorter frontal (f) - abfrontal (a) length. mc: mucocyte. **D, E.** Asymbiotic specimen. TEM of cells in the abfrontal zone of a gill filament. Note the absence of bacteria. The epithelium is pseudostratified, with apical cells (c2) overlying basal cells (c1) containing large mitochondria (m). mv: microvilli.

Out of the 76 specimens from which we had corresponding morphological and molecular data, we observed two dramatically different gill filament morphologies. Most specimens (N = 55) had opaque, pink to white gills, with ‘type 3’ filaments as described in [4] (Fig. 2a). These gills were abfrontally expanded, with a clear bacteriocyte zone abfrontal to the frontal ciliated zone, and TEM observations revealed large numbers of extracellular symbionts (Fig. 2b). In contrast, 21 individuals had thin, translucent ‘type 2’ gills, conspicuously lacking a bacteriocyte zone (Fig. 2c). The abfrontal epithelium was pseudostratified, with apical cells (c2) overlying more basal cells (c1), the latter containing numerous enlarged mitochondria (Fig. 2d, e). Among those individuals with type 2 gills, we saw no symbiotic bacteria and very little toluidine blue staining (apart from mucocytes) in all sections observed.

In post-hoc analyses, we were unable to distinguish symbiotic and asymbiotic individuals based on the structure of any organ besides the gills, which generally appeared thicker and more opaque in symbiotic individuals. We noticed that the ferruginous patch on the posterior end of the shell was often located further dorsally in symbiotic individuals than in asymbiotic ones.

The shell size of symbiotic individuals (3.25±0.095 mm) was significantly greater than that of asymbiotic individuals (2.50±0.077 mm) [F(1, 75) = 3.97, p = 1.25E-05]. However, the prodissoconch length of symbiotic (average ± SD: 200±23 μm, N = 18) and asymbiotic (average ± SD: 193±9 μm, N = 7) individuals did not differ significantly (t-test, p>0.25).

### Molecular descriptions: OTUs

18S fragments (size range: 960 -1001 bp) were successfully sequenced from 104 individuals, all most closely matching *Thyasira gouldi* from Mill Bay, Salcombe, UK (GenBank accession number JF899224) with a sequence similarity of 99%. In 84 cases, corresponding 28S fragments (range: 1232 – 1448 bp) were successfully sequenced, all most closely matching *Thyasira gouldi* from Mill Bay, Salcombe, UK (JF899196) with a slightly lower sequence similarity (98%). We defined three distinct Operational Taxonomic Units (OTUs) in which 3 sites (out of 1001) and 13 sites (out of 1448) were polymorphic within the 18S and 28S fragments, respectively (Table 1). Individuals grouping together for the 18S fragment also grouped together for the 28S fragment. GenBank accession numbers are in Table 2.

**Table 1:** Polymorphic nucleotide sites within sequenced 18S and 28S gene fragments of *Thyasira* cf. *gouldi* OTUs.

OTU	Collection site	18S			28S												
		229	233	283	729	746	773	781	788	790	797	824	885	971	990	995	1026
1	D(9), N(21), S(37)	C	C	T	C	C	T	G	C	T	A	C	T	C	C	T	C
2	D(3), N(5), S(1)	.	T	.	T	.	.	A	T	C	G	.	A	A	T	C	T
3	D(19), S(9)	T	T	A	T	T	C	A	.	.	.	A	A	A	T	C	T

The number of individuals of each OTU sequenced from each collection site (Deer Arm, D; Neddy's Harbour, N; Southeast Arm, S) is indicated in parentheses. Dots indicate the same base as in the top row.

**Table 2:** Accession numbers, *Thyasira* cf. *gouldi* OTU 18S rRNA, 28S rRNA and CO1 gene fragments.

OTU	18S	28S	CO1
1	KJ424346	KJ424347	KJ424337, KJ424338, KJ424339
2	KJ424348	KJ424349	KJ424340, KJ424341, KJ424342
3	KJ424350	KJ424351	KJ424343, KJ424344, KJ424345

Further confirmation of OTU groupings was obtained through mitochondrial CO1 sequences. We successfully obtained CO1 sequences (range: 256–667 bp) from 75 of the above-mentioned 104 individuals; closest matches in GenBank corresponded to *Thyasira obsoleta* (AM706507, 84% similarity) and *T. ferruginea* (AM706499, 83% similarity). Although the CO1 sequences were variable, there was strong support for three groups, corresponding exactly to OTUs 1, 2 and 3 defined by 18S & 28S sequences (100% posterior probability (PP) based on Bayesian reconstruction [data not shown]).

Based on the 76 specimens from which we had corresponding gill ultrastructure data, all individuals from OTUs 1 (N = 49) and 2 (N = 6) were symbiotic, while all individuals from OTU 3 (N = 21) were asymbiotic.

The molecular divergence values within and between pairs of OTUs (*d*), based on CO1, are in Table 3. The one-way ANOVA comparing *d* values within and between OTUs was statistically significant [F(2, 2340) = 115144, p<0.0001]. We did not observe any major violations of ANOVA assumptions. The Tukey's HSD post-hoc test revealed significant differences when comparing within and between OTU *d* values, average within-OTU *d* being one order of magnitude lower (range: 0.00071 - 0.01116) than average between-OTU *d* (range: 0.1152 to 0.1178) (Table 3).

**Table 3:** Distance matrix based on CO1 (667 bp) OTU sequences.

	Between OTUs		Within OTUs
	OTU 1	OTU 2	
**OTU 1**			0.00071±0.00020^c^
**OTU 2**	0.11714±0.01128^a^		0.00150±0.00082^c^
**OTU 3**	0.11779±0.01184^a^	0.11519±0.01175^b^	0.01116±0.00227^d^

*d* values (averages) were calculated using the ‘p-distance’ model in MEGA5, treating gaps/missing data with pairwise deletion. Standard error estimates on each comparison are based on 2000 bootstrap replicates. Significant differences (p<0.01) based on Tukey's HSD post-hoc test are indicated by different letters (^a–d^).

### Shell shape analysis

**Figure 3:**
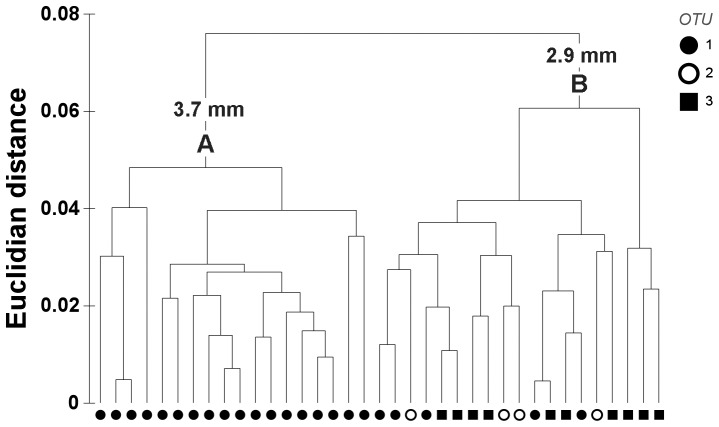
Cluster analysis of shell outlines (Elliptical Fourier Analysis). The analysis is based on the first 10 harmonics. Shells form two groups, A and B, which differ significantly in length (indicated above the groups) based on a t-test (p<0.01). Symbols refer to operational taxonomic groups (OTUs) based on CO1, 18S and 28S rRNA sequences.

The first cluster analysis isolated two groups at a distance of 0.075 (Fig. 3). The first group (A) contained only symbiotic individuals (18 OTU 1) whereas the second group (B) included all three OTUs (5 OTU 1, 4 OTU 2, 10 OTU 3). Specimens from group A were larger than those from group B (t-test, p<0.01, Fig. 3).

**Figure 4:**
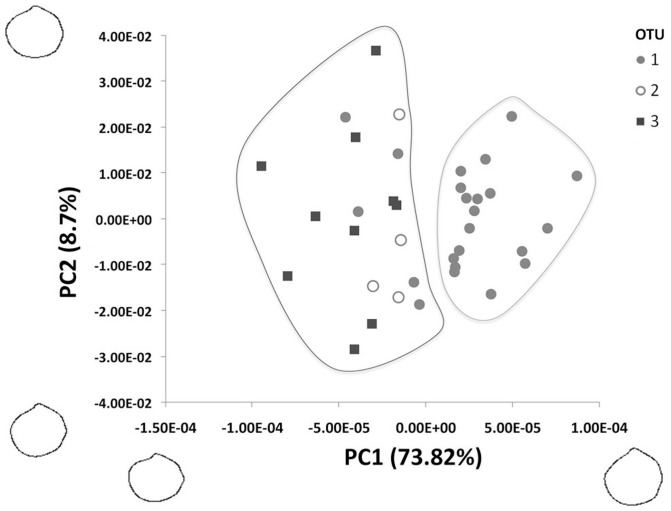
Shape variation among shells along the first two principal components. Reconstructed contours show shapes at the extremities of each axis. Symbols refer to operational taxonomic groups (OTUs) based on CO1, 18S and 28S rRNA sequences.

The first PCA, based on the 10 first harmonics (40 values per shell), provided 4 significant principal components (PC). PC1 represented at least 73% of the variability observed (Fig. 4), and two groups of individuals could be separated along this axis (corresponding to groups A and B in Fig. 3). Reconstruction of outlines at both extremities of this axis showed differences in the length/height ratio and anterior curvature, with specimens from group A characterized by a more equilateral ovate polygonal outline and specimens from group B being more subequilateral subovate (Fig. 4; terminology as in [13]). The second axis explained only 8.7% of the variability, based upon the curvature of the anterior-ventral margin; symbiotic and asymbiotic specimens could not be discriminated along this axis.

**Figure 5:**
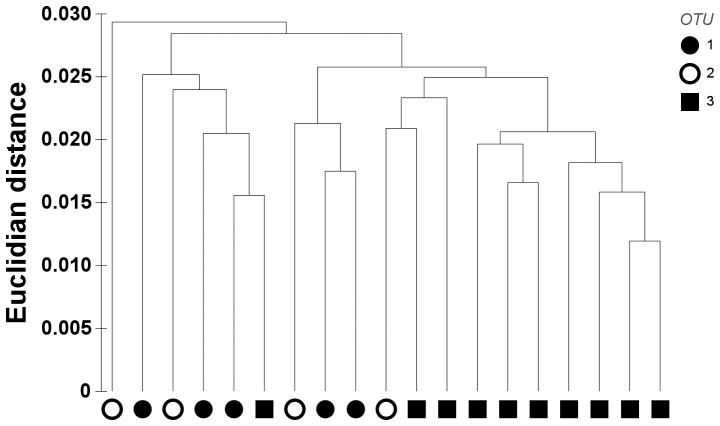
Cluster analysis of a subset of shell outlines (Elliptical Fourier Analysis). Shells analysed are those from group B in Figure 3. The analysis is based on the first 20 harmonics, excluding the first one. Symbols refer to operational taxonomic groups (OTUs) based on CO1, 18S and 28S rRNA sequences.

**Figure 6:**
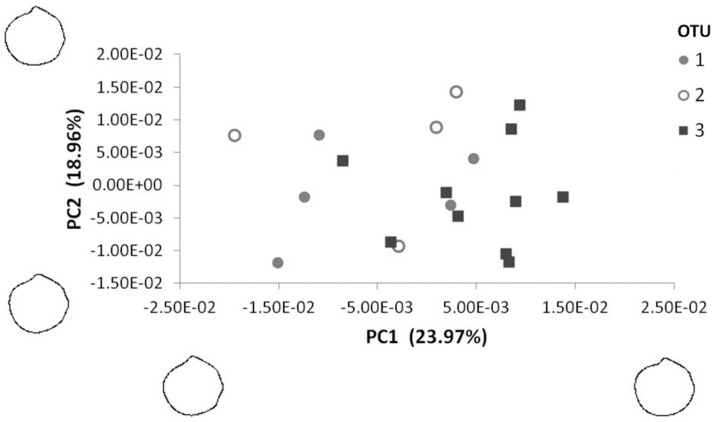
Shape variation in a subset of shells along the first two principal components. Shells analysed belong to group B in Figure 3. Reconstructed contours show shapes at the extremities of each axis. Symbols refer to operational taxonomic groups (OTUs) based on CO1, 18S and 28S rRNA sequences.

A second analysis performed on specimens from group B (20 harmonics, with the first one removed) revealed 11 significant PCs. The cluster on the PCA values grouped most OTU 3 individuals together at a distance of 0.02 (Fig. 5); ANOSIM results indicated significant differences between OTU groups (R = 0.39, p<0.01), with greater separation between OTUs 1 and 3 (R = 0.40) and OTUs 2 and 3 (R = 0.52) than between the symbiotic OTUs 1 and 2 (R = −0.04). PC1 explained 23.97% of the observed variability, and outline reconstructions showed that symbiotic individuals tend to have a more sinuate posterior margin, a more pronounced anterior-ventral expansion, and a more sloping lunule margin than asymbiotic individuals, which were more rounded and had a more pronounced beak (Fig. 6). Along PC2 (18.95% of variability), symbiotic and asymbiotic individuals could not be separated.

### Geographic distribution

**Figure 7:**
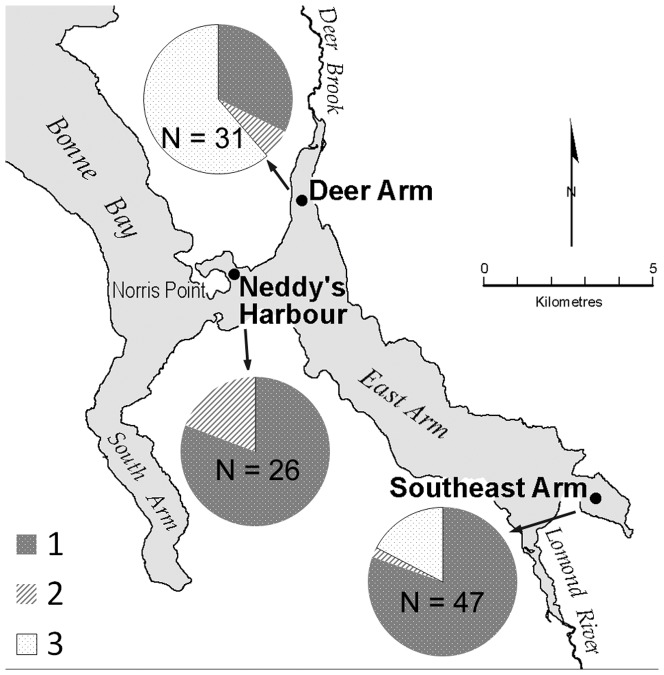
Distribution of *Thyasira* cf. *gouldi* groups in Bonne Bay, NL. Charts at each location show relative proportions of each OTU (1–3) and total number of individuals (N) used in the molecular analysis. Sampling sites are Deer Arm, Neddy's Harbour, and Southeast Arm.

Within Bonne Bay, symbiotic individuals were found at all three sampling sites. Asymbiotic individuals were slightly more common than symbiotic individuals in Deer Arm, were relatively rare in Southeast Arm, and were absent altogether in Neddy's Harbour (Fig. 7).

## Discussion

### Species identification

All specimens in this study could be assigned to the species *Thyasira gouldi* based on shell characteristics, including: 1) an equilateral-ovate outline, 2) a well-defined submarginal and posterior fold, 3) the presence of an auricle, and 4) a narrowly rounded ventral margin, appearing slightly angulate in several of the specimens [11]. As in other thyasirids [1], shell outline is thought to be highly variable in this species [11]; this was observed in our sampling. Moreover, the large prodissoconch (i.e., readily visible by eye) observed in the Bonne Bay specimens is a key characteristic of *Thyasira gouldi* [11], [15]. This identification is further confirmed by 18S and 28S gene sequences, which are most similar to those of *Thyasira gouldi* from Mill Bay, Salcombe, UK. However, because of consistent differences in several of the studied characters, as detailed below, we remain uncertain about the species determination of Bonne Bay specimens and designate them as *Thyasira* cf. *gouldi* until further testing of species delineation hypotheses can be done.

### Differences in symbiosis-related characters

Our examination of over 150 *T*. cf. *gouldi* specimens from Bonne Bay revealed a high degree of disparity in examined characters among specimens. Most remarkable was the finding of both symbiotic and asymbiotic individuals within this group, at each sampling date, and often within very close proximity (i.e., the same grab). To our knowledge, this is the first report of such a highly divergent symbiotic condition among bivalves appearing to belong to a single species.

The differences observed did not only concern symbiont abundance, as previously documented in *Thyasira equalis* [4] and produced experimentally in *T. flexuosa* [9]. Rather, we observed striking and consistent differences in gill morphology (frontal-abfrontal thickness) that were not the result of differences in sectioning angle. In post-larval bivalves with homorhabdic filibranch gills, the frontal-abfrontal thickness of filaments remains relatively constant as gill filaments differentiate [34], [35]. Our specimens had either gill type 2 or 3 [4], regardless of their size (> 1 mm shell length; we did not study smaller individuals). Gill type was also clearly associated with symbiont presence: there were no specimens with symbiotic type 2 gills, or asymbiotic type 3 gills. Those observations suggest that symbionts play important roles in modulating the development of this structure. In the symbiotic clam *Codakia orbicularis*, the frontal-abfrontal thickening of gill filaments (associated with the development of bacteriocytes) is triggered by exposure to their environmentally-transmitted symbionts during the juvenile stage [36]. Similarly, the early postembryonic development of the symbiotic organ of the squid *Euprymna scolopes* is controlled by bacterial symbionts [37], and rates of proliferation of zebrafish intestinal epithelial cells are regulated by the resident microbiota [38]. The possibility that thyasirid symbionts might similarly influence the development of gill filaments is intriguing and merits further study.

Symbiotic specimens attained a larger body size than asymbiotic individuals, probably as a result of greater trophic efficiency in the former group. The additional nutritional input gained from symbionts was hypothesized to be the cause of larger body size in symbiotic compared to asymbiotic juveniles of *Codakia orbicularis* [36]. Comparing reported sizes of symbiotic and asymbiotic thyasirids reveals a similar trend [4].

Asymbiotic and symbiotic *Thyasira* cf. *gouldi* were found in close proximity (i.e., sometimes within the same grab sample); this distribution might be related to sulphide patchiness at the sampling sites. At a cold seep, the distribution of two chemosymbiotic vesicomyids (*Calytogena kilmeri* & *C. pacifica*) with different sulphide physiology characteristics was related to the fine-scale patchiness of sulphide [39]. Environmental characteristics may also explain the apparent absence of asymbiotic *T*. cf. *gouldi* at Neddy's Harbour, the shallow site that has coarser sediments, experiences higher temperature and salinity variations (unpublished data) and is subject to dredging (R. Hooper, pers. comm.).

### Differences in shell characters

Shell shape differed, although imperfectly, between symbiotic and asymbiotic *Thyasira* cf. *goudi* specimens. In a first cluster analysis, symbiotic individuals with dorso-ventrally elongated shells (mainly larger specimens) were clearly distinct from other individuals (a mixture of smaller symbiotic and asymbiotic specimens). Smaller symbiotic individuals were similar in dorso-ventral length to asymbiotic specimens, but slight differences in shell outline appeared between those groups. The extent of dorso-ventral elongation of thyasirid shells is thought to be associated with burrowing depth and motility in sediments: species with shells higher than long are deep burrowers while species with shells longer than high remain close to the sediment surface [40]. It could be advantageous for symbiotic *T.* cf. *gouldi* to burrow more deeply since the reduced sulphur compounds presumably required for the chemoautotrophic metabolism of their symbionts would be more accessible in deeper sediment layers. Also, the slightly more dorsal location of the ferruginous patch on the shell of symbiotic *T*. cf. *gouldi* may be associated with a physiological and behavioural adaptation to symbiosis: the location of this patch appears to be associated with the location of inhalant and exhalant currents [40]. Comparing the ventilation, burrowing and sulphur mining [41], [42] activities of symbiotic and asymbiotic *T*. cf. *gouldi* could help explain observed differences in shell characters within this group.

We found no difference in the prodissoconch size of asymbiotic and symbiotic *Thyasira* cf. *gouldi*. The size range obtained was fairly large, and some uncertainty in sizing is likely due to the angle of the shell when imaging took place. The prodissoconch measures between 205−270 μm in *T. gouldi* individuals (N = 55) from Norway, Faeroe Islands, New England and Greenland [11], but is smaller in Bonne Bay specimens (180 − 210 μM, N = 25). Prodissoconch size can vary with latitude, with size increasing from south to north [11]. The factors influencing prodissoconch size in thyasirids, and the usefulness of this character in species identification, require further attention.

### Differences in nuclear and mitochondrial gene sequences

Genes bring further support for the presence of distinct groups among the specimens studied. We identified three OTUs based on nuclear and mitochondrial gene sequences, and evolutionary *d* in the CO1 gene sequences between pairs of OTUs was significantly higher than the *d* within OTUs suggesting the co-occurrence of three distinct (cryptic) species in Bonne Bay: two symbiotic and one asymbiotic. We are currently unable to distinguish OTUs 1 and 2 based on morphology or ultrastructure. Until these groups are formally described, we retain their OTU designation.

A previous study of four undescribed *Thyasira* sp. individuals reported very low levels of divergence (<0.1%) within the 28S gene fragment and no difference in 18S fragments [43]. In comparison,18S and 28S genes in *T. gouldi* show a high degree of variation: for example, 18S sequences of *T. gouldi* from the north and south coasts of the UK (AJ581871 and JF899224) have 8 bp discrepancies within 956 bp, and 28S sequences from those same sites differ by 12 bp out of 573. Therefore, the *T. gouldi* lineage appears highly divergent.

### Possible species complex within *Thyasira gouldi*

Because of: 1) consistent differences in shell outline, gill characters and gene sequences between symbiotic and asymbiotic Bonne Bay individuals; 2) the presumed amphi-Atlantic geographic range of this larval brooding [44] species; 3) the considerable variation in shell outline reported for this species [11]; 4) the high degree of variability in nuclear and mitochondrial gene sequences between specimens from different locations, we argue that *Thyasira gouldi* forms a cryptic species complex. As most of the information currently available on type specimens consists of shell characters (descriptions of internal anatomy and gene sequences only apply to specimens from the eastern Atlantic), a detailed examination and genetic analysis of material from the type location will be required to properly delineate species within this group.

Cryptic, sibling, or incipient species complexes [45]–[48]) have been discovered in the chemosymbiotic bivalve families Vesicomyidae [49], [50] and Lucinidae [51], but not in the Thyasiridae. However, unlike in these cases, the *T. gouldi* complex is remarkable as it comprises both symbiotic and asymbiotic species. A clear relationship between OTU and symbiont presence was found, as all OTU 1 and OTU 2 individuals were symbiotic and had elongated (type 3) gill filaments, while all OTU 3 individuals lacked symbionts and had short (type 2) gill filaments. Thus, symbiosis may constitute a particularly strong evolutionary driving force in this group, with symbiont presence and gill structure being subject to change over relatively short evolutionary timescales. Further phylogenetic analyses including *T. gouldi* specimens from other sites should help elucidate the sequence of symbiont appearance (or loss) within this group. The relatively rapid switch between symbiotic and asymbiotic states observed here suggests that symbionts are not vertically transmitted but are acquired from the sedimentary environment (i.e. horizontally or environmentally) at a juvenile stage, as described in other invertebrates [52], [53]. Under this scenario, and considering that gill filament elongation is triggered by symbionts, it is conceivable that groups of thyasirids subjected to different environmental pressures would either associate with symbionts or not. Reproductive isolation in different groups would be facilitated by the small-scale dispersion of brooded juveniles and eventually lead to speciation.

## Conclusions

Many gaps remain in understanding the evolutionary history and distribution patterns within the Thyasiridae, especially since published gene sequences for most thyasirid species represent a single geographic location, and possibly a single individual. Species-level identification of thyasirids based on shell features is challenging because of the paucity of clear diagnostic characters that are often confounded by convergent or parallel evolution [25], and the presumed high intraspecific variation in shell form [1]. The wide distribution of many thyasirids should be re-assessed in light of the differences observed here in gene sequences from apparent conspecifics spanning the Atlantic Ocean. The striking differences we show in gill filament morphology underscore the relevance of this organ as a species diagnostic character [40]; symbiosis can directly affect gill morphology and have significant (and possibly rapid) ecological and evolutionary consequences for the host.
